# Atlanta Academy of Medicine

**Published:** 1879-03

**Authors:** H. F. Scott

**Affiliations:** Reporter


### ATLANTA ACADEMY OF MEDICINE.

---

H. F. SCOTT, M.D., REPORTER.

---

ATLANTA, GA., February 10, 1879.

President Dr. J. S. Tood, in the chair.

Dr. Salm desired to present the synopsis of a case demonstrating the utter futility of treating chancroid with mercurials and caustics.

On the 10th of January last, Mr. Z. came to him and stated that he had been under the treatment of several physicians, one of whom had given him mercurials and applied caustics to the ulcer for about two weeks, but the latter steadily growing worse, he had gone to another physician, who recommended mercurial inunction, and besides gave mercurials internally, and with local application of caustics, but all to no purpose.

On examining his penis, he found one of the most marked cases of chancroid, that had ever come under his observation—being a deep ulcer upon summit of glans, running along corona, upon right side, to fraenum into urethra; also a fistulous opening from bed of ulcer; there was no swelling of lymphatics.

Patient stated that the case was of three month's standing, and was afraid glans penis would slough away. In order to cleanse ulcer of muco-purulent discharge, and set up a healthy action, he prescribed the following lotion, to wit:

| | |
|---|---|
| Hydrary. bichlor corros, - - | 0, 40 g |
| Spir. vini concen, - - - - | 4, 00 g |

to be applied three or four times daily, with camel's hair pencil, and covered with charpie.

On his return, two day's later, we had a fair view of the case, and prescribed:

| | |
|---|---|
| Ferri Potass-Tart, - - - | 15, 00 g |
| Aqua pura, - - - - - - | 250, 00 g |

Tablespoonful four times daily; few drops of same fluid to be applied to ulcer several times a day; enjoining, at the same time, scrupulous cleanliness.

A few days thereafter, he returned with no improvement, except closure of the aforementioned fistulous opening; the treatment was, nevertheless, persevered in, and, at the expiration of about five days, ulcer began to show signs of healthy granulations, with decrease of œdema and hyperæmia, in surrounding tissues.

In addition to above treatment, he was now given the following:

| | |
|---|---|
| Argenti Nitratis, - - - - - | 0, 48 |
| Glycerrhi pura, - - - - | 60, 00 g |
| Aqua, distill, - - - - - - | 60, 00 g |

to be dropped three or four times daily, on ulcer.

The last lotion was three days later, rendered more concentrated by the addition of twelve centigrammes, (two grains) of nitrate of silver.

Under this treatment, depression left by ulcer had about half filled out, and his patient was of "*good cheer.*"

February 5th.—Found marked improvement, but changed treatment to:

| | |
|---|---|
| Ferri sesquichlor, - - - - - | 2, 00 g |
| Glyc. pura., - - - - - | 2, 00 g |
| Spirit vini concen, - - - - - | 1, 00 g |
| Aqua. distill, - - - - - - | 1, 00 g |

to be applied with camel's hair pencil.

February 10th.—Saw patient, and was doing remarkably well.

The President asked Dr. Salm the length of time patient had been under his treatment, and expressed opinion that treatment pursued above was virtually using caustics. Said all admitted that mercurials were not indicated in chancroid.

Dr. Salm replied that the case had been under his treatment for four weeks, and was not yet entirely healed. Is of the opinion that any local applicant should be varied from time to time, as it was apt to lose its effect.

Dr. Wilson is an advocate of caustics, from favorable result in his hands. Related a case where glans penis being almost entirely destroyed, and wishing to save as much as possible, he applied nitric acid, and followed it by a poultice. He afterwards applied nitrate of silver, and prescribed tonics. He likewise treated a young female with several chancroids just within labia minora. Both cases progressed favorably.

Dr. Calhoun hasn't had much recent experience, but should be tempted to use caustics, to say the least. Thought cauterization would not aggravate the case; but, on the contrary, hasten the healing process.

Dr. Howell related a case of Dr. Willis Westmoreland's, where the application of nitric acid to severe chancroid was used with fine result.

The President said it was not now fashionable to cauterize chancroid; but at least in phagadenic chancroid they should be applied with a vim. On the day after cauterizing he was accustomed to sprinkle on powdered iodoform, which was preferable to a salve of the same, from not being so offensive an odor. This powder relieves the pain and promotes healthy granulation.

Dr. Mack believes in cauterizing chancroid, and has usually found it efficient, if thoroughly applied; where the caustic doesn't readily penetrate to the seat of the disease, it is only necessary that it be repeated. In one case where he had cauterized a phagadenic ulcer patient neglected to return for inspection, and the glans sloughed entirely away. The consequent bleeding and ulcer were best relieved with cauterizing iron, heated to a dull redness. This, together with tonics and food, cured the case. The hot iron acted as hæmostatic and local stimulant.

Dr. Calhoun presented two specimens of enucleated eyeballs, with history of the cases. In the first case, that of a boy, aged 10, a piece of unexploded cap had struck the upper lid, just under the eyebrow; wound bled temporarily, and was examined by a physician, who detected no foreign body. Next day, patient feeling no unpleasantness, went out hunting. During the day the ball began to swell, and in six or eight days, when he saw the patient, it was of enormous size and very hard, and pus was to be seen in anterior chamber—in fact, it presented every appearance of panophthalmitis. Thinking that a piece of the cap was within the eye, he decided upon enucleatio bulbi, which was accordingly done. Upon making the last cut the slight pressure caused pure pus to be poured out through the wounded lid, and with it the cap, which had penetrated the lid and eyeball and set up the inflammation and immense infiltration of surrounding tissues. Patient recovered in a short time. Peculiar, in that the boy saw naturally, and presented no untoward symptoms till 48 hours subsequent to injury. There had been no symptom of sympathetic ophthalmia

The other case presents several interesting aspects. An old lady, 68 years of age, while in attendance upon her daughter during confinement, had charge of the baby, which was suffering from ophthalmia neonatorum of so severe a natnre as to result in the loss of both eyes. Accidentally getting some of the virus in her own eye, inflammation of a severe type ensued; cornea became infiltrated and ulcerated, and entirely sloughed eventually, leaving iris exposed and bulging forward. Vision became daily worse, and symptoms of sympathetic ophthalmia beginning to manifest themselves, in the other eye, he, at patient's request, removed the stump of the ball. The removal was rendered the more difficult from the absence of cornea, and narrowness of the interpalpebral fissure, due to infiltration of the parts; hence the slightest pressure sufficed to cause loss of humors. Dr. C. said the case was mainly interesting from the age of the patient and manner of its contraction. In children it was well known that purulent ophthalmia was almost always fatal to the sight. In answer to a question from the President as to orthodox treatment of ophthalmia neonatorum, Dr. Calhoun stated that on account of the excessive secretion of pus, much stress should be laid on the importance of cleanliness, which should be attended to every half hour, the bad results in many cases being due to a lack of proper cleanliness.

The attending physician should use once daily a solution of nitrate of silver, 10 grains to an ounce of water, neutralizing with salt water. The attendants should be given a weak solution of sulphate of zinc, with directions to apply it freely.

The President related a case where a woman, washing her eyes with urine for sore eyes, innoculated them with gonorrhœal virus. In this case he used a 20-grain solution of nitrate of silver, but found it too strong; nevertheless, patient recovered.

Dr. Calhoun heard of a remedy to-day for the first time —it being the use of fresh cow-dung poultice in sore eyes.

Several members mentioned its use by the laity in various affections.

Dr. Wilson was sent for by a young lady, aged 22, who had been sick since last June; she complained of frequent micturition, somewhat bloody, and of loss of flesh and appetite. Had been treated in Chattanooga for cystitis and nephritis with blisters, parsley tea, etc; had also been treated for retroflexion of the uterus. On examination he found womb in normal position, but sensitiveness about urethra, in which he could only introduce a No. 8 French bougie; it seems there was a stricture half an inch from posterior to meatus, following a severe gonorrhœa, which the patient acknowledged having had. Having administered ether, he introduced a "meatrome" with exceeding difficulty and divided the stricture to the extent of allowing the introduction of a No. 36 bougie. Gave quinine and hip-bath, and secured comparatively good night's rest. The patient was an opium eater, and knowing that the antidote she was using contained this article, he ordered it in increased doses, and 5 or 6 grains quinine daily. Had operated on the 24th January; Saturday after the patient was very much better; gave flax seed tea to relieve irritation about the bladder.

Dr. Mack asked Dr. Wilson his treatment for prostatitis in aged persons. He replied: Blister to the perineum, leeches and warm hip-bath.

Dr. Mack stated that ergot was used by Dr. Cabbott, of Boston, with great benefit; he gives it in ½ to 1 drachm doses of the fluid extract, carefully watching its effects. Recommended placing the "eye" of a pair of scissors on the lids and pressing backwards so as to surround and fix firmly the eye-ball, in cases where foreign bodies, such as bits of iron and steel had become impacted in cornea. By this method, for the suggestion of which he was indebted to a friend, one was enabled to safely remove such objects without any anæsthetic or the use of a speculum. Asked the members which was more commonly given in Atlanta —ether or chloroform?

Dr. Calhoun uses chloroform invariably and without any dread of its effects; where children are unruly, he often gives it to quiet them during an examination or treatment of the eyes; never had any trouble to follow, except in one case, and which he succeeded in overcoming. Some patients would shudder at the sight of scissors, hence he uses the fingers alone, placing one upon the upper and another upon the lower lid, and pressing firmly backwards so as to effectually protrude the ball and steady it between the two fingers.

Dr. Mack said ether was used almost exclusively in Boston, and chloroform was very much in prejudice, so much so that one losing a case during its administration, would not be sustained by the courts.

Billworth having lost two cases from chloroform, quite a sensation was produced, and since then, B. had usually given a mixture of ether and chloroform.

In conversation with Dr. Squibbs, he was told that Squibbs' ether was made exactly according to the Pharmocopea; he thought they wasted ether in Boston, where they frequeutly gave a patient an immense quantity. Said that the more of it that was given the greater the likelihood of vomiting ensuing; hence, he (Squibbs) had invented an inhaler by which he could keep one anæsthetized $1\frac{1}{2}$ hours with only 2 ounces ether.

The President said the difficulty with inhalers that he had inspected, was that too little air could enter them, and asked if he didn't consider them dangerous on that account.

Dr. Mack said statistics did not indicate this; besides, they should be taken away at intervals.

The President mentioned that Drs. Willis Westmoreland and J. T. Johnson used ether exclusively.

Dr. Calhoun said the objection to ether was the amount consumed and the length of time necessary to its administration; besides, there was a great waste, which filled the room and clothes of the operator. Besides, nine out of ten times it was followed by vomiting, which was disagreeable always, and particu'arly dangerous after cataract oprations.

Dr. Scott was opposed to the indiscriminate use of chloroform; he usually gives ether ålone, or, in cases where nausea would be prejudical, he begins with ether until he has tested the patient's ability to stand an anæsthetic, and then finishes up with chloroform.

By pursuing this course he had never had any disagreeable consequences to ensue, and all nausea was, as a rule, obviated.

In operating for cataract, he generally gave no anæsthetic at all; whilst in enueclatio bulbi he preferred ether, as the attendant nausea was rather beneficial than otherwise.

Mentioned that Bosey, of Berlin, used an injection into rectum of fluid extract ergot and glycerine, for enlarged prostate; whilst others injected hypodermically in perineum.

Academy adjourned.

---

January 20, 1879.

The President J. S. Todd, M.D., in the chair.

Dr. A. W. Calhoun was called recently to LaGrange,

Georgia, to see Clifford Mayson, a youth, aged ten, who had met with a painful accident, which resulted in the loss of an eye.

While running with a two edged knife in his hand, the blade being about two inches long, he fell, the blade entering the socket, at the inner canthous, making a wound at right angles with the lid. Upon its withdrawal by patient, a wound nearly at right angle with nose was produced, and also the ball was entirely dislocated, but not wounded. The force of the fall was so great that the blade of the knife was bent. All efforts by the attending physicians to replace the ball were futile.

When he arrived, the following history and treatment was given him by the medical attendants:

Failing to restore the eye to its place, they had kept emollient applications to the face to prevent sloughing of the cornea, but dispite this proper procedure, the ball had become soft, and the cornea already—twenty hours after accident—showed signs of sloughing. In a few hours after the receipt of the injury, the pulse fell to between thirty and forty, and the respiration accordingly short. He had never lost consciousness, and complained of but little pain. It was seriously feared that he would sink, as they believed the brain had been entered through the roof of orbit. In five or six hours, however, he rallied, and was, so far as pulse, breathing, etc were concerned, in a natural condition when seen first by Dr. C. He found the ball tightly held by the lid, and completely extruded from socket. After an hour's manipulation, he succeeded in restoring it to its place, but so soon as pressure was removed it would immediately pop out again. A careful examination of the orbit with finger, revealed no solution of the continuity of the bones. He said that the depression aforementioned, was occasioned by compression; the pressure being caused by the forcible dragging forward of that portion of the brain which gives origin to the optic nerves, and that as the brain accommodated itself to the new relations of the parts, the depression wore off. Under chloroform, the eye was enucleated in the ordinary way. The optic nerve was cut close up to the ball, and immediately retracted. It was found, upon examination of ball, that the internal rectus muscle was severed, and also, half the circumference of the optic nerve, and with the central artery of the retina. The extravasation of blood, which must have taken place immediately from the cut artery, has filled the orbit, and this prevented the replacement in the first place, and the absent muscle allowed the displacement to recur as often as the globe was replaced.

The protruded ball completely hid the lids, and to inspect them and the parts adjacent, was the object of Dr. C. in replacing the eye, before enucleating it.

Dr. Baird was of the opinion that shock, and not pressure, was the cause of the depression. A large nerve was wounded, and much force used, much more than in ordinary enucleation. The President asked Dr. C. if enucleation of the eye, the patient not being under chloroform, was usually followed by such depression, and if there were other evidences of shock, weak pulse, moist skin, etc.? Dr. C. said that he had never performed the operation without first anesthetizing patient, but that the history of such cases before chloroform or ether was used, give no account of shock. The cutting of the nerve is comparatively harmless. There were no other symptoms of shock, the pulse, while slow, was full and hard, and the skin natural.

The President agreed with Dr. C., and thought that a restoration of the pulse and breathing to a normal condition, was facilitated not only by the parts accustoming themselves to the changed relations of things, but also to the fact, that the natural elasticity of the nerve allowed of the part pulled forward in a measure resuming its accustomed place.

---

February 24, 1879.

The Academy called to order by the President, Dr. J. S. Todd.

No reporter's abstract.

Dr. Calhoun presents a cockleburr as specimen, and relates case of a boy æt. 11, who, in driving up cows a week ago yesterday evening, ran through field with mouth open and received several of the burrs into his mouth. His breathing instantly became very difficult, while he introduced his finger and took out two or three of the offending objects. The breathing was, however, not at all improved by the removal of these, and immediately that he reached home a physician was called. The parents were advised to send the boy to Dr. C., and he was brought to him the next day.

When Dr. C. saw him his breathing was extremely labored, and he could neither speak out nor whisper. Examination with the laryngoscope revealed a burr lying between the two vocal cords, and the parts very much swollen. The burr was so firmly held that after repeated trials at extraction with laryngeal forceps tracheotomy seemed inevitable. Fortunately, on a last attempt, the offending body came away. Instantly the breathing improved, and the boy, who had not slept since the accident occurred, fell into a refreshing sleep in the office chair; went to sleep again at 2 o'clock and slept until 12 o'clock the day following. Examination next day revealed ulceration of vocal cords. The child did very well afterwards, and was sent home in a few days.

Heard from him yesterday, but has not yet recovered power of speech.

The President resigning the chair in favor of Dr. Calhoun, related a case of a boy who had likewise gotten a cockleburr into his bronchi, but without being conscious of it. Cough, accompanied by profuse expectoration and other signs of phthisis, colliquative sweats, etc., followed, for which reason patient was supposed to be suffering from tuberculosis. After remaining in his bronchi for 2 years it was finally dislodged and discharged during a profuse expectoration of pus and mucus. Followed by complete recovery.

Dr. Low knew of a case where a boy retained a nickel in his larynx for a long period of time. Cough and expectoration existed during whole time. Finally thrown off, and coin was found to be sharpened on edge.

Dr. Miller knew of a case where a cockleburr had been lodged in air-passages, but was fatal in 48 hours.

Dr. Calhoun cited the case of a boy who, when 7 years of age, had some foreign body enter his air-passages. For seven years longer he was constantly troubled, and no one had hopes of his recovery. At the end of these seven years Dr. C. saw him. He could distinctly hear the rattling in his trachea, as if some foreign body moved up and down with respiration. Examination with laryngoscope revealed nothing. Saw no more of him for two years, when he was called in consultation. The physical signs indicated pus in pleura. And after procuring the additional consultation of Dr. Battey, they proceeded to introduce the trocar and canula. An immense quantity of pus was evacuated at the time. The opening was somewhat enlarged for the evacuation and injecting of the cavity, but during a severe fit of coughing it served the better purpose of giving exit to the long-offending body—a cockleburr.

The exciting cause of this attack was exposure to cold atmosphere after confinement in a warm room.

The patient soon recovered and is now a strong, healthy young man, exhibiting his burr as a curiosity.

Dr. Calhoun next related a case of extirpation of ball of eye. The patient, a young man, suffered about five years ago from iritis and cyclitis at same time. Lymph was thrown out on the crystaline lens as a result and gave it the appearance of a cataract on casual observation.

On examining eye some three months ago found that some softening had taken place and advised removal of ball. The patient objected and returned home. A few days ago he came back complaining of pain in the other eye, and upon examination found commencing sympathetic inflammation and ophthalmia. Again advised extirpation, and patient acquiesing, the operation was performed. Has been doing very well since that time.

The President desired to know the result of the administration of digitalis in the hands of the different members, and also the doses given.

Dr. Salm stated that he had given the tincture in 10 gtt. doses.

Dr. Cortelyon had taken Squibbs' fl. ext. in 10 gtt.

doses every four hours. Reduced the frequency of his pulse very perceptibly. Alternated the digitalis with aconite. Had used it afterwards in 10 gtt. doses, and always reduced frequency of pulse. Did not notice marked effect on urine.

Dr. Miller gave it formerly very frequently. Lately has confined its use to cases of kidney disease. Gives tinct. in 10 gtt. doses, continued for short periods only, as he thinks there is danger in its administration for long continued periods. Cites the case of an old man with hydrothorax to whom he had given digitalis with great relief. The following year it was prescribed by some one else in larger doses and for a longer time and the patient died from effects of medicine.

Dr. Conally has a patient with cardiac valvular trouble to whom he gave digitalis in 2 gtt. doses once or twice a day. No good result followed, when he increased the dose to 5 gtt., since which time the improvement has been marked.

About a year ago he had a case of anasarca in which he administered digitalis, but with no effect. Diuretic action of drug marked in present case; not marked in one of year ago.

The President asked Dr. Miller if he thought the digitalis was absorbed in those cases of delirium tremens where drachm doses of the tincture were administered? Dr. Miller thought not, but gave as his opinion that full doses of *time* were the best remedy in that disease.

The President then asked if the danger from the exhibition of the remedy had not passed when the diuretic action was once produced? Dr. M. does not use it after he obtained that result, that being the object of his giving it.

Dr. Salm said, that the question of the advantages and the disadvantages of the use of iodoform had been discussed at the last meeting, and that the odor had been thought to be the greatest disadvantage. Had seen the following formula in a journal purporting to obviate that odor:

| | | |
|---|---|---|
| ℞—Iodoform, gram., | - - - - | 2 |
| Faseline, | " - - - - - | 30 |
| Olie Amaki, | - - - - - | 6 gtt. |

Dr. Tood said that patient would rebel against use of above, as pain equalled that of nitric acid.

Dr. Low asked the result of treatment of epilepsy by iron, quinine and strychinne.

Dr. Salm instanced case in his practice where the convulsions had been relieved up to present twice by this treatment, combined with the bromides.

Dr. Low related case successfully treated.

Dr. Miller knows of several cases, and thinks it a curable disease, but not by medicine.

Upon motion, the Academy adjourned.

